# Left ventricular mechanical dispersion in flow-gradient patterns of severe aortic stenosis with narrow QRS complex

**DOI:** 10.1007/s10554-019-01754-y

**Published:** 2020-01-13

**Authors:** Daniel Lavall, Linn Kristin Kuprat, Joscha Kandels, Stephan Stöbe, Andreas Hagendorff, Ulrich Laufs

**Affiliations:** Klinik und Poliklinik für Kardiologie, Universitätsklinikum Leipzig, Liebigstrasse 20, 04103 Leipzig, Germany

**Keywords:** Aortic stenosis, Low-flow, Strain, Mechanical dispersion, LV dysfunction

## Abstract

Patients with severe aortic stenosis are classified according to flow-gradient patterns. We investigated whether left ventricular (LV) mechanical dispersion, a marker of dyssynchrony and predictor of mortality, is associated with low-flow status in aortic stenosis. 316 consecutive patients with aortic stenosis and QRS duration < 120 ms were included in the retrospective analysis. Patients with severe aortic stenosis (aortic valve area ≤ 1.0 cm^2^) were classified as normal-flow (NF; stroke volume index > 35 ml/m^2^) high-gradient (HG; mean transvalvular gradient ≥ 40 mmHg) (n = 79), NF low-gradient (LG) (n = 62), low-flow (LF) LG ejection fraction (EF) ≥ 50% (n = 57), and LF LG EF < 50% (n = 23). Patients with moderate aortic stenosis (aortic valve area 1.5–1.0 cm^2^; n = 95) served as comparison group. Mechanical dispersion (calculated as standard deviation of time from Q/S onset on electrocardiogram to peak longitudinal strain in 17 left ventricular segments) was similar in patients with NF HG (49.4 ± 14.7 ms), NF LG (43.5 ± 12.9 ms), LF LG EF ≥ 50% (47.2 ± 16.3 ms) and moderate aortic stenosis (44.2 ± 15.7 ms). In patients with LF LG EF < 50%, mechanical dispersion was increased (60.8 ± 20.7 ms, p < 0.05 vs. NF HG, NF LG, LF LG EF ≥ 50% and moderate AS). Mechanical dispersion correlated with global longitudinal strain (r = 0.1354, p = 0.0160) and heart rate (r = 0.1587, p = 0.0047), but not with parameters of aortic stenosis. Mechanical dispersion was similar among flow-gradient subgroups of severe aortic stenosis with preserved LVEF, but increased in patients with low-flow low-gradient and reduced LVEF. These findings indicate that mechanical dispersion is rather a marker of systolic myocardial dysfunction than of aortic stenosis.

## Introduction

Aortic stenosis (AS) is the most prevalent valvular heart disease in industrialized countries [1]. In patients with severe aortic stenosis, mortality increases with the onset of symptoms. Timely aortic valve (AV) replacement restores normal life expectancy [2]. Severe AS is determined by reduced aortic valve area (AVA) and increased AV pressure gradient and velocity on echocardiography [3, 4]. However, diagnosis of severe AS can be challenging because up to 1/3 of patients present with discordant findings between AV pressure gradient and AVA [5, 6]. The current guidelines recommend—after the exclusion of measurement errors—a classification into subgroups according to flow, gradient and left ventricular (LV) ejection fraction (EF) for therapeutic management and prognostic considerations [7, 8].

Particularly, the pathophysiology of the low-flow (stroke volume index ≤ 35 ml/m^2^) severe aortic stenosis is incompletely understood. Several reasons have been proposed such as pronounced LV concentric remodeling, impaired diastolic filling, reduced LV systolic longitudinal function, contractile dysfunction, and high afterload burden [6, 9]. Speckle-tracking echocardiography allows detailed analysis of LV mechanics. Global longitudinal strain (GLS) has emerged as robust parameter of LV function and allows detection of subclinical LV dysfunction despite normal LVEF [10, 11]. Mechanical dispersion (MD), which is calculated as standard deviation of the time-to-peak longitudinal strain in standardized myocardial segments of the LV, is a measure of intraventricular dyssynchrony [12, 13]. A higher variation of the time-to-peak strain, i.e. increased mechanical dispersion, reflects heterogeneous LV contraction that predicts arrhythmic risk [14] and might impair LV systolic performance. While complete bundle branch block causes obvious LV dyssynchrony [15], the hemodynamic impact of MD in patients with narrow QRS complex (≤ 120 ms) is unknown.

We investigated whether mechanical dispersion is associated with low-flow status in aortic stenosis and narrow QRS (≤ 120 ms) complex. LV mechanical dispersion was measured retrospectively in patients with severe aortic stenosis according to flow-gradient patterns in a large university hospital echocardiography database.

## Methods

### Patients

**Fig. 1:**
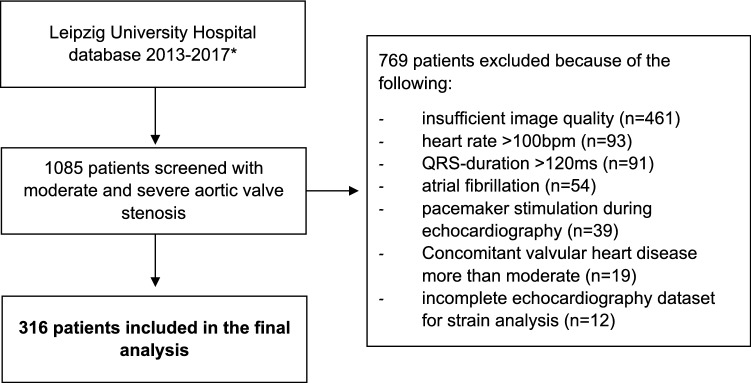
Flow chart of the study. The figure demonstrates the extraction of patients with moderate and severe aortic stenosis from the hospital data warehouse, reasons for exclusion, and the final study population. *2015–2017 for patients with moderate aortic stenosis

Echocardiographic data warehouse of a tertiary care hospital (Universitätsklinikum Leipzig) was screened for patients with the diagnosis “aortic stenosis” between 2013 and 2017. Consecutive patients with severe (AVA ≤ 1.0 cm^2^) and moderate (AVA 1.0 to 1.5 cm^2^) aortic stenosis according to continuity equation [4] were included. Exclusion criteria were > moderate mitral or tricuspid valve disease, > moderate aortic valve regurgitation, pacemaker stimulation during echocardiography, heart rate > 100 bpm, hypertrophic cardiomyopathy, bundle branch block (QRS duration > 120 ms), atrial fibrillation without similar R–R cycles as well as insufficient image quality precluding strain analysis. Since high quality images of echocardiography are necessary to obtain valid strain measurements, strict exclusion criteria regarding image quality have to be applied. Hence, the number of patients per group does not represent the true prevalence of aortic stenosis subtypes. The study flow chart is shown in Fig. 1. Patients with moderate aortic stenosis served as comparison group and were consecutively included from 2015 to 2017.

Patients with severe aortic stenosis were divided into 4 groups according to the current guidelines [3, 4]:

- Normal-flow (stroke volume index > 35 ml/m^2^) and high-gradient (AV mean pressure gradient ≥ 40 mmHg) (=NF HG)
- Normal-flow, low-gradient (AV mean pressure gradient < 40 mmHg) (=NF LG)
- Low-flow (stroke volume index ≤ 35 ml/m^2^), low-gradient and left ventricular ejection fraction (LVEF) ≥ 50% (=LF LG EF ≥ 50%), i.e. paradoxical low-flow, low-gradient AS
- Low-flow, low-gradient and left ventricular ejection fraction (LVEF) < 50% (=LF LG EF < 50%), i.e. classical low-flow, low-gradient AS

The clinical parameters, i.e. heart failure symptoms, blood pressure, medication, comorbidities and ECG, were extracted from the hospital patient management data warehouse. The study was approved by the local ethics committee (No. 119/18-ek).

### Echocardiography

Standard echocardiography with 2D, Doppler and tissue Doppler imaging were performed according to current standards using Vivid E9 and E95 (General Electric, Horton, Norway). Quantitative analysis was conducted offline with the EchoPac software (General Electric, Horton, Norway). LV end-diastolic and end-systolic diameter, LV mass and relative wall thickness (RWT) were determined from M-mode in the parasternal short axis view [11]. Body surface area, estimated with Dubois formula, was used for indexation of echocardiographic parameters. LV end-diastolic (LVEDVi) and end-systolic volume index (LVESVi) were calculated according to the 2D biplane method of discs [11]. LVEF was calculated as (LVEDVi-LVESVi)/LVEDVi. Stroke volume index (SVi) was determined from pulsed-wave Doppler and diameter of the LV outflow tract and body surface area [4]. Cardiac index (CI) was calculated as SVi × heart rate.

Peak E-wave velocity, peak A-wave velocity, and the E/A ratio was determined from pulsed-wave Doppler at the mitral valve inflow [16]. E’ was measured at the septal and lateral mitral valve using tissue Doppler, and E/E’ was calculated from a mean E’ value [16].

AV peak velocity and mean AV pressure gradient were measured from continuous-wave Doppler through the aortic valve in the apical long axis view [4]. Severe aortic stenosis was confirmed by planimetry of AVA on transesophageal echocardiography or by low-dose dobutamine stress echocardiography, as appropriate [3]. The valvulo-arterial impedance (Zva), a measure of global LV afterload in aortic stenosis integrating valvular and arterial load [17], was calculated as the sum of systolic arterial blood pressure and the mean AV pressure gradient, divided by SVi. Aortic regurgitation was graded according to the recent recommendations using a multi-parametric approach [18].

### Deformation analysis

**Fig. 2:**
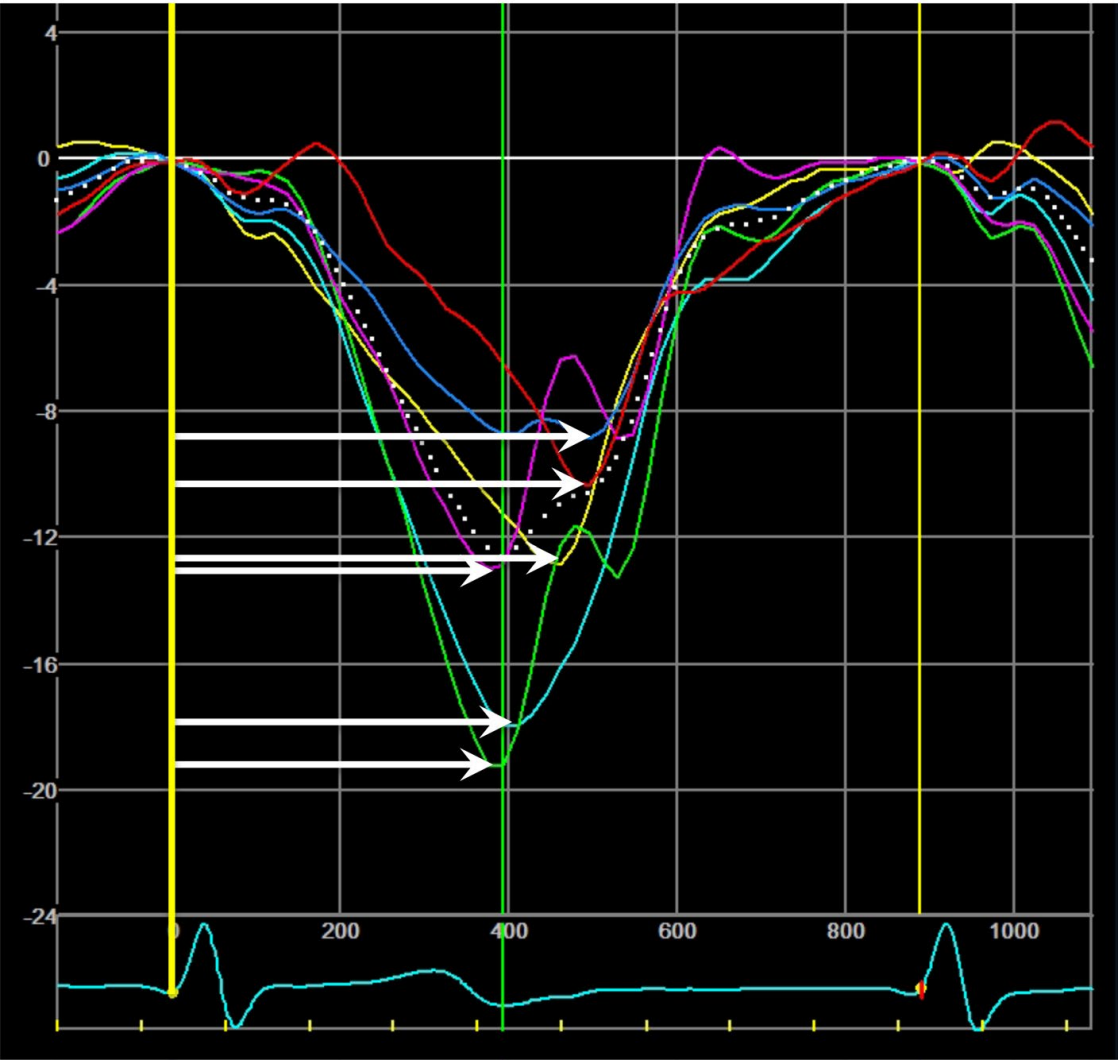
Measurement of mechanical dispersion from longitudinal strain analysis. Time-to-peak strain (horizontal arrows) was measured in each myocardial segment derived from 2D speckle-tracking echocardiography. The yellow vertical line represents the beginning of Q/S, i.e. the systole. Mechanical dispersion was calculated as the standard deviation of time-to-peak strain in 17 myocardial segments

Global longitudinal peak systolic strain (GLS) was derived from speckle-tracking analysis of apical 4-chamber-, 2-chamber- and long-axis views according to the 17 segment LV model [11]. Systolic time interval was determined from LV outflow tract pulsed-wave Doppler. Time-to-peak strain was measured as time from Q/R onset on ECG to peak longitudinal strain in each LV segment (Fig. 2). If more than two myocardial segments could not be analyzed (e.g. due to poor image quality or a complete lack of a negative strain curve), the patient was removed from the analysis. Mechanical dispersion was calculated as the standard deviation of time-to-peak strain values in 17 LV segments [12]. Heart rate was measured from simultaneous ECG during echocardiography on bulls-eye plot of GLS. To account for different heart rates in the study population, MD was additionally normalized to a heart rate of 60 bpm (i.e. to a cycle length of 1000 ms). This analysis was performed by calculating the normalized time-to-peak-strain values in each of the 17 LV myocardial segments with subsequent calculation of a normalized MD value. In atrial fibrillation, strain analysis was performed in apical views with similar cycle lengths allowing GLS and MD measurements.

### Intra- and inter-observer variability

Intra- und inter-observer variability for GLS and MD was assessed in 30 randomly selected patients. The intra-observer variability was determined by blinded re-analysis (L.K.K.) after a time period of at least 8 weeks from the initial analysis. Interobserver variability (L.K.K. vs. D.L.) was assessed by blinded analysis. The intra-observer correlation coefficient for GLS was r = 0.958 (95% confidence interval (CI) 0.911–0.981, p < 0.0001), mean difference 0.2 ± 1.1% (p = 0.414), and for MD r = 0.879 (95% CI 0.754–0.943, p < 0.0001), mean difference 0.5 ± 5.8 (p = 0.556) for MD. The inter-observer variability was for GLS r = 0.903 (95% CI 0.800–0.954, p < 0.0001), mean difference 0.7 ± 1.8% (p = 0.09), and for MD r = 0.846 (95% CI 0.5692–0.926, p < 0.0001), mean difference 0.8 ± 7.5 (p = 0.543).

### Statistical analysis

Data are presented as mean ± standard deviation or percentages, as appropriate. One-way ANOVA test with correction for multiple comparisons (Tukey test) was applied for statistical comparisons of continuous variables between multiple groups. Categorical variables were compared using Chi-square test, providing a global p value.

The correlation coefficient was calculated using the Spearman method. Intra- und interobserver variability was analyzed using Spearman correlations and the Wilcoxon matched-paired test. Statistical analysis was performed with GraphPad Prism software, version 6. Statistical significance was considered with a two-sided p < 0.05.

## Results

### Patient characteristics

The characteristics of the 316 patients are shown in Table 1. Age, NYHA functional class, blood pressure and cardiovascular comorbidities were similar among patients with different flow-gradient patterns of aortic stenosis. Those with LF LG EF < 50% were on similar heart failure medications than the other groups despite suffering from reduced LVEF. QRS duration was higher in patients with LF LG EF < 50% compared to the other groups of aortic stenosis.

**Table 1:** Clinical characteristics

Clinical characteristics	NF HG N = 79	NF LG N = 62	LF LG EF ≥ 50% N = 57	LF LG EF < 50% N = 23	Moderate N = 95	P value
Age (years)	74.1 ± 11.3	73.9 ± 10.9	78.8 ± 9.3^*^	76.7 ± 9.8	72.1 ± 10.8^§^	0.0043
Male sex (%)	55.7	45.2	36.8	73.9	52.6	0.0262
BMI (kg/m^2^)	26.5 ± 4.3	26.2 ± 4.0	27.0 ± 4.3	25.5 ± 3.4	26.9 ± 4.1	ns
NYHA functional class III/IV (%)	42.86	48.78	45.00	58.33	41.54	ns
Systolic blood pressure (mmHg)	137.8 ± 22.8	143.6 ± 22.0	140.3 ± 26.9	138.6 ± 23.2	141.7 ± 24.1	ns
Diastolic blood pressure (mmHg)	78.7 ± 15.7	75.0 ± 11.4	76.2 ± 13.9	79.2 ± 19.1	77.5 ± 13.3	ns
Hypertension (%)	77.8	68.6	78.2	57.1	78.0	ns
Diabetes (%)	25.4	19.6	29.1	19.0	31.7	ns
Dyslipidemia (%)	55.6	56.9	54.5	57.1	56.1	ns
Coronary artery disease (%)	41.3	33.3	30.9	38.1	30.5	ns
Previous myocardial infarction (%)	7.9	9.8	5.5	23.8	14.6	ns
Previous PCI (%)	7.9	25.5	12.7	14.3	18.3	ns
Previous CABG (%)	4.8	5.9	5.5	4.8	2.4	ns
Renal impairment (%)	32.8	21.2	50.9	57.1	30.1	ns
GFR (ml/min/1.73m^2^)	66.8 ± 21.3	70.3 ± 19.3^§^	58.1 ± 22.8^†^	58.6 ± 25.8	66.5 ± 4.8	0.0398
Beta blocker (%)	50.0	59.6	72.7	57.1	60.0	ns
Calcium channel blocker (%)	31.3	36.5	25.5	19.0	35.0	ns
ACE-I/ARB/ARNI (%)	73.4	69.2	76.4	47.6	76.3	ns
MR antagonists (%)	3.1	3.8	7.3	19.0	3.8	ns
Diuretics (%)	42.2	34.6	63.6	61.9	43.8	0.0165
Other antihypertensives (%)	6.3	11.5	14.5	14.3	8.8	ns
Ivabradine (%)	0	3.8	0	0	0	0.0742
Antiarrhythmics (%)	3.1	1.9	1.8	0	0	ns
Digitoxin (%)	4.7	1.9	3.6	4.8	6.3	ns
QRS duration (ms)	88.1 ± 12.9	86.5 ± 12.9^||^	84.2 ± 15.2^||^	101.8 ± 13.4^*†‡§^	87.1 ± 11.5^||^	0.0009
Atrial fibrillation (%)	17.5	19.6	45.5	19.0	28.0	0.0056

Since normal-flow (NF; stroke volume index > 35 ml/m^2^), high-gradient (HG; AV mean pressure gradient ≥ 40 mmHg) was considered as reference group of aortic stenosis, statistical symbols were not displayed the NF HG column. Renal impairment was defined as estimated glomerular filtration rate (eGFR) < 60 ml/min/1.73m^2^. ACE-I, angiotensin converting enzyme inhibitor; ARB, angiotensin II receptor type 1 blocker; ARNI, angiotensin receptor neprilysin inhibitor; BMI, body mass index; CABG, coronary artery bypass graft; EF, ejection fraction; LF, low-flow (stroke volume index ≤ 35 ml/m^2^); LG, low-gradient (AV mean pressure gradient < 40 mmHg); MR, mineralocorticoid receptor; PCI = percutaneous coronary intervention. * p < 0.05 vs. moderate aortic stenosis (AS); † p < 0.05 vs. NF LG; ‡ p < 0.05 vs. NF HG; § p < 0.05 vs. LF LG EF ≥ 50%; || p < 0.05 vs. LF LG EF < 50%

Echocardiographic parameters of LV dimensions, LV function and aortic stenosis are presented in Table 2. NF HG was considered as the reference groups for severe aortic stenosis. Due to the subgroup definitions, AV velocity and pressure gradients, stoke volume index, LVEF and aortic valve area differed between the groups. Both patients with LF LG EF ≥ 50% and LF LG EF < 50% exhibited elevated valvulo-arterial impendance compared to the other groups. Prevalence of concomitant moderate aortic regurgitation was < 20% in all groups. Patients with LF LG EF ≥ 50% were characterized by smaller end-diastolic LV volume but high relative wall thickness and similar GLS compared to NF HG. Patients with LF LG EF < 50% exhibited increased LV end-diastolic and end-systolic volumes with lower relative wall thickness, reduced GLS, low cardiac index despite higher heart rate, and an increased E/A ratio.

**Table 2:** Echocardiographic data

	NF HG N = 79	NF LG N = 62	LF LG EF ≥ 50% N = 57	LF LG EF < 50% N = 23	Moderate N = 95	P value
Heart rate (HR) (bpm)	71.5 ± 12.0	69.2 ± 10.6^§||^	76.1 ± 11.9^†^	79.5 ± 14.7*^†‡^	70.8 ± 11.1^||^	0.0004
LV end-diastolic diameter (mm)	44.9 ± 6.1	43.5 ± 5.7^||^	42.6 ± 7.3^||^	52.5 ± 8.3*^†‡§^	45.3 ± 6.7^||^	< 0.0001
LV end-systolic diameter (mm)	28.9 ± 4.9	27.0 ± 5.6^||^	27.4 ± 6.7^||^	41.0 ± 7.5*^†‡§^	29.2 ± 6.0^||^	< 0.0001
LV mass index (g/m^2^)	160.8 ± 46.3	134.0 ± 31.8^‡||^	130.2 ± 36.5^‡||^	172.1 ± 51.9*^†§^	127.8 ± 44.7^‡||^	< 0.0001
RWT	0.68 ± 0.15	0.65 ± 0.15	0.69 ± 0.20*^||^	0.56 ± 0.18^‡§^	0.58 ± 0.16^‡§^	< 0.0001
LV end-diastolic volume index (ml/m^2^)	58.1 ± 18.0	49.4 ± 12.1^‡||^	48.0 ± 15.1^‡||^	75.7 ± 27.5*^†‡§^	54.2 ± 15.8^||^	< 0.0001
LV end-systolic volume index (ml/m^2^)	22.4 ± 10.5	18.3 ± 6.6^||^	20.1 ± 8.1^||^	50.0 ± 19.65*^†‡§^	20.3 ± 8.3^||^	< 0.0001
LV EF (%)	62.7 ± 7.8	63.5 ± 7.2^||^	59.1 ± 7.1*^†||^	33.5 ± 8.3*^†‡§^	63.0 ± 7.8^§||^	< 0.0001
Stroke volume index (ml/m^2^)	44.9 ± 6.4	43.6 ± 6.1^§||^	28.5 ± 4.4*^†‡^	26.5 ± 6.0*^†‡^	42.8 ± 11.2^§||^	< 0.0001
Cardiac index (l/min/m^2^)	3.2 ± 0.6	3.0 ± 0.6^§||^	2.2 ± 0.4*^†‡^	2.0 ± 0.4*^†‡^	3.0 ± 08^§||^	< 0.0001
E/A	0.9 ± 0.5	1.0 ± 0.7^||^	1.0 ± 0.8^||^	1.6 ± 1.2*^†‡§^	1.0 ± 0.4^||^	0.0128
E/E'	18.6 ± 7.4	17.9 ± 9.3	17.9 ± 8.2	20.9 ± 7.7	15.8 ± 7.2	ns
Peak aortic valve jet velocity (m/s)	4.9 ± 0.6	3.7 ± 0.4*^‡§||^	3.3 ± 0.6^†‡^	3.2 ± 0.6^†‡^	3.1 ± 0.8^†‡^	< 0.0001
Mean aortic valve gradient (mmHg)	56.8 ± 14.6	30.2 ± 5.9*^‡||^	25.9 ± 8.0^‡^	22.6 ± 8.6^†‡^	23.0 ± 12.3^†‡^	< 0.0001
Valvulo-arterial impedance (mmHg/ml/m^2^)	4.4 ± 0.8	4.1 ± 0.8^§||^	6.0 ± 1.4*^†‡^	6.8 ± 3.5*^†‡^	4.1 ± 1.2^§||^	< 0.0001
Aortic valve area (cm^2^)	0.71 ± 0.14	0.9 ± 0.17*^‡§||^	0.75 ± 0.12*^†^	0.74 ± 0.19*^†^	1.2 ± 0.19^†‡§||^	< 0.0001
Aortic valve area index (cm^2^/m^2^)	0.39 ± 0.08	0.5 ± 0.08*^‡§||^	0.42 ± 0.07*^†^	0.41 ± 0.1*^†^	0.65 ± 0.12^†‡§||^	< 0.0001
Moderate aortic regurgitation (%)	19.0	8.1	14.0	13.0	10.5	ns
GLS (%)	− 18.6 ± 3.8	− 19.9 ± 2.8^§||^	− 16.9 ± 3.4*^†‡||^	− 8.6 ± 3.7*^†‡§^	− 19.0 ± 3.9^§||^	< 0.0001
Mechanical dispersion (ms)	49.4 ± 14.7	43.5 ± 12.9^||^	47.2 ± 16.3^||^	60.8 ± 20.7*^†‡§^	44.2 ± 15.7^||^	< 0.0001
Mechanical dispersion normalized to 60 bpm (ms)	57.9 ± 16.5	49.9 ± 15.9^||^	59.0 ± 20.2^||^	82.9 ± 36.6*^†‡§^	52.0 ± 20.3^||^	< 0.0001

Since NF HG was considered as reference group of aortic stenosis, statistical symbols were not displayed for this group. *GLS* global longitudinal strain, *LV* left ventricular, *RWT* relative wall thickness; other abbreviations as in Table 1. *p < 0.05 vs. moderate aortic stenosis (AS); ^†^p < 0.05 vs. NF LG; ^‡^p < 0.05 vs. NF HG; ^§^p < 0.05 vs. LF LG EF ≥ 50%; ^||^p < 0.05 vs. LF LG EF < 50%

### Mechanical dispersion

**Fig. 3:**
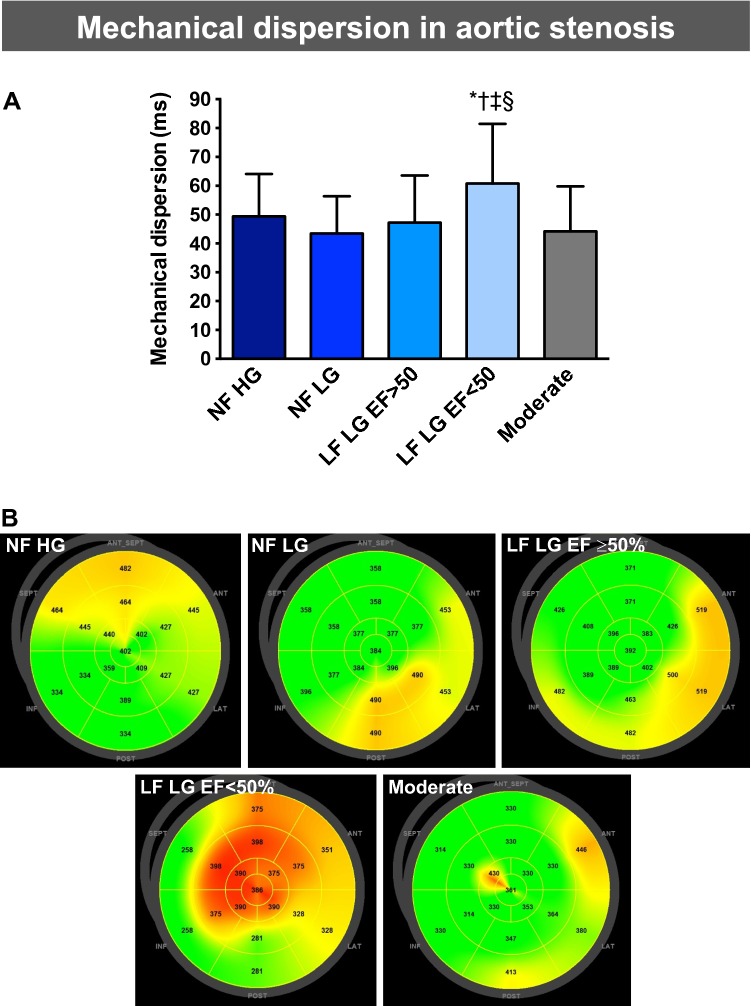
Summary figure of mechanical dispersion in aortic stenosis. **a** quantitative comparison of mechanical dispersion in subgroups of aortic stenosis. **b** representative LV bulls-eye plots with color-coded time-to-peak strain values for each myocardial segment. *EF* ejection fraction, *HG* high-gradient (AV mean pressure gradient ≥ 40 mmHg), *LF* low-flow (stroke volume index ≤ 35 ml/m^2^), *LG* low-gradient (AV mean pressure gradient < 40 mmHg), *NF* normal-flow (stroke volume index > 35 ml/m^2^). *p < 0.0001 vs. moderate aortic stenosis; ^†^p < 0.0001 vs. NF LG; ^‡^p < 0.05 vs. NF HG; ^§^p < 0.01 vs. LF LG EF ≥ 50%

Left ventricular mechanical dispersion is shown in Fig. 3. Between NF HG (49.4 ± 14.7 ms), NF LG (43.5 ± 12.9 ms), LF LG EF ≥ 50% (47.2 ± 16.3 ms) and moderate (44.2 ± 15.7 ms) AS, there was no difference in mechanical dispersion (Table 2). Mechanical dispersion in patients with LF LG EF < 50% (60.8 ± 20.7 ms) was increased compared to NF HG (p = 0.0177), NF LG (p < 0.0001), LF LG EF ≥ 50% (p = 0.0043) and moderate AS (p < 0.0001; Fig. 3). Since the different heart rate between the groups may impact strain values, MD was normalized to a heart rate of 60 bpm. By this method, the results were confirmed: There were no differences of MD between NF HG (57.9 ± 16.5), NF LG (49.9 ± 15.9 ms), LF LG EF ≥ 50% (59.0 ± 20.2 ms) and moderate (52.0 ± 20.3 ms) AS. MD of LF LG EF < 50% patients (82.9 ± 36.6 ms) was increased compared to compared to NF HG, NF LG, LF LG EF ≥ 50% and moderate AS (p < 0.0001 for all) (Table 2).

### Association of mechanical dispersion with aortic stenosis and LV function

**Fig. 4:**
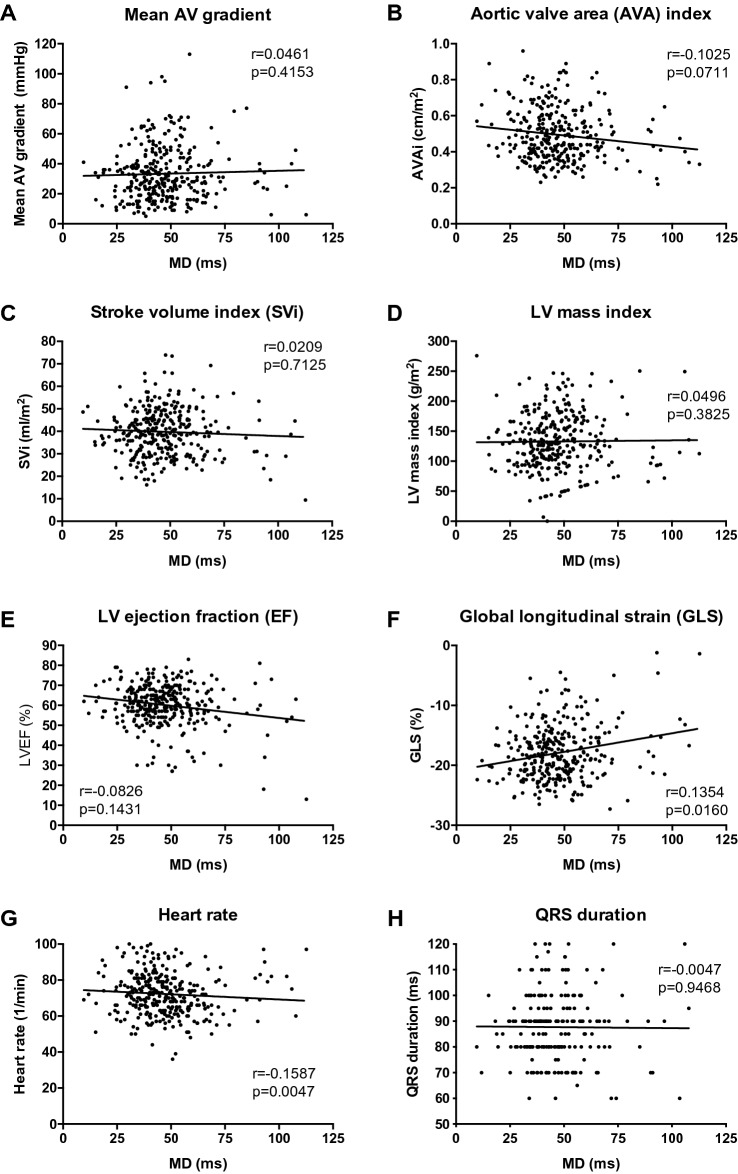
Correlations of mechanical dispersion. Mechanical dispersion (MD) and **a** mean aortic valve (AV) gradient, **b** aortic valve area (AVA) index, **c** stroke volume index (SVi), **d** LV mass index, **e** LV ejection fraction (EF), **f** longitudinal systolic strain (GLS), **g** heart rate, and **h** QRS duration. Linear regression lines, correlation coefficients (r) and p values are presented in the figure

To evaluate hemodynamic associations of MD, a correlation analysis with parameters of aortic stenosis, LV remodeling, LV systolic function and QRS duration was performed. In the entire cohort of patients with aortic stenosis, there was no correlation of MD with mean AV gradient, AVA index, LV mass index (Fig. 4), valvulo-arterial impedance (r = 0.0265, p = 0.648, data not shown), LVEDVi (r = − 0.0217, p = 0.7017, data not shown), LVESVi (r = 0.0393, p = 0.4873, data not shown), stroke volume index, LVEF or QRS duration (Fig. 4). There was a weak but significant correlation of MD with GLS and with heart rate (Fig. 4).

We performed an additional explorative correlation analysis by including patients with chronic systolic heart failure (n = 84 consecutive patients, mean LVEF 35 ± 7%, MD 59.4 ± 16.7 ms, extracted from the echocardiography database) without aortic stenosis to account for a full spectrum of LV remodeling and LV function. In this population, MD correlated significantly with LVEDVi (r = 0.1804, p = 0.0003), LVESVi (r = 0.2530, p < 0.0001), LVEF (r = − 0.2895, p < 0.0001) and GLS (r = 0.3108, P < 0.0001).

## Discussion

Our study demonstrates that mechanical dispersion is similar among flow-gradient subgroups of severe aortic stenosis with preserved LVEF. Patients with low-flow, low-gradient aortic stenosis and reduced LVEF (< 50%) showed increased mechanical dispersion, i.e. intraventricular dyssynchrony despite having a narrow QRS complex. These data indicate that mechanical dispersion is marker of LV systolic dysfunction, rather than a distinct characteristic of aortic stenosis.

Since severe aortic stenosis is a heterogenous entity, concise hemodynamic characterization is of particular importance. We considered normal-flow high-gradient (NF HG) as the reference group because these patients fulfill all criteria for severe aortic stenosis [3, 4]. Patients with normal-flow, low-gradient (NF LG) represent an intermediate stage between moderate and NF HG severe aortic stenosis [6, 19], reflected by intermediate values of LV mass, relative wall thickness, valve area, and AV gradients in our study. Paradoxical low-flow low-gradient (LF LG EF ≥ 50%) aortic stenosis patients are characterized by advanced concentric LV hypertrophy with high relative wall thickness, high valvulo-arterial load, small LV volumes and thus reduced stroke volume despite preserved LVEF [6, 19, 20]. Patients with classical low-flow low-gradient (LF LG EF < 50%) are characterized by eccentric LV hypertrophy and impaired systolic LV function, i.e. reduced LVEF, GLS, stroke volume and cardiac index. In addition, classical LF LG patients had advanced diastolic dysfunction indices, increased heart rate and high valvulo-arterial load. Thus, patients classical LF LG represent with a complex hemodynamic situation of systolic heart failure with an additional afterload burden due to severe aortic stenosis.

Mechanical dispersion (MD) is derived from LV longitudinal strain analysis and represents intraventricular dyssynchrony of contraction [12, 14, 21, 22]. Our data show that in patients with severe aortic stenosis and preserved LVEF, mechanical dispersion was similar whether flow was low or normal. In particular, patients with paradoxical LF LG aortic stenosis do not exhibit subclinical LV dyssynchrony despite their low-flow situation. These data indicate that mechanical dispersion is not a hemodynamic determinant of low-flow state per se in aortic stenosis if LVEF is preserved. In contrast, increased mechanical dispersion in patients with dilated left ventricles and impaired systolic LV function, i.e. classical LF LG aortic stenosis, indicates that these patients suffer from LV dyssynchrony even in the absence of a bundle branch block. These data indicate that LV dyssynchrony seems to be insufficiently represented in the surface ECG, setting the stage for incremental value of MD in patients with narrow QRS complex. Patients with bundle branch block, i.e. QRS duration > 120 ms, were excluded from the analysis because they have obvious LV dyssynchrony [15].

Myocardial fibrosis is one of several underlying factors of increased MD [21]. A recent study found increased myocardial fibrosis in patients with classical LF LG aortic stenosis compared to patients with HG aortic stenosis with preserved LVEF, which might represent the morphological substrate for increased MD in this group [23]. Furthermore, heart rate may impact values of mechanical dispersion, which should be taken into account when comparing strain measurements. Correlation analysis revealed no associations between MD and parameters of aortic stenosis severity in our study. Prihadi et al. reported a correlation of MD with LV mass, aortic valve area, age, LVEF and QRS duration [13]. These discrepancies might be attributable to dissimilar study populations with up to 50% of patients with bundle branch block or intraventricular conduction abnormalities in the aforementioned study [13]. Taken together, bundle branch block occurs frequently in severe AS and is a cause for increased MD. Subclinical LV dyssynchrony in patients with severe AS and narrow QRS complex seems to be related to the underlying LV dysfunction rather than to valvular load due to aortic stenosis. These data are supported by an exploratory analysis including patients with chronic systolic heart failure without aortic stenosis showing significant associations between MD and parameters of LV remodeling and LV dysfunction. However, these hypothesis-generating findings have to be confirmed in further studies.

MD is a robust parameter as shown by good inter- and intra-observer variability in our analysis and by others [12, 24]. MD has been identified as a marker for the occurrence of ventricular arrhythmias in numerous cardiac diseases [14], such as after myocardial infarction [12, 22] and in hypertrophic cardiomyopathy [21]. Klaeboe et al. demonstrated a prognostic implication of MD for predicting all-cause mortality in patients with severe aortic stenosis, which was incremental to LVEF, GLS and valvulo-arterial impedance [24]. A recent retrospective analysis in patients with various degrees of aortic stenosis confirmed the prognostic value of MD for all-cause mortality and reported that MD increases with the severity of disease [13]. However, these studies did not analyze mechanical dispersion in the subgroups of aortic stenosis according to flow and gradient [3, 6]. Our analysis characterized MD in subtypes of aortic stenosis with narrow QRS complex according to flow, gradient and LVEF. The data argue against a subclinical LV dyssynchrony based on MD measurement in patients with severe AS in both normal-flow and low-flow states as long as LVEF is preserved. Those with LF LG and reduced LVEF had increased mechanical dispersion indicating LV dyssynchrony. This finding support the well-established prognostic impact of MD because these patients had the worst prognosis in the literature [6].

## Limitations

This is a retrospective analysis with its inherent limitations. NYHA function class assessment might have poor accuracy due to the lack of complete and systematic data acquisition. The aim of this mechanistic study was to evaluate the hemodynamic associations of MD in patients with aortic stenosis. Therefore, prognostic implications of MD were beyond the scope of our study. Whether MD provides incremental prognostic information in aortic stenosis subgroups has to be evaluated by further prospective studies. There are several reasons for reduced LVEF in patients with classical LF LG aortic stenosis and in those with chronic heart failure, hence a causal association with MD cannot be proven based on these data.

## Conclusion

In patients with severe aortic stenosis and narrow QRS complex, mechanical dispersion was similar in low-flow and normal-flow subgroups if LVEF was preserved. Patients with low-flow low-gradient aortic stenosis and reduced LVEF had increased mechanical dispersion, i.e. pronounced intraventricular dyssynchrony. These data indicate that mechanical dispersion is rather a marker of systolic myocardial dysfunction than of aortic stenosis.

## References

[1] Nkomo VT, Gardin JM, Skelton TN (2006). Burden of valvular heart diseases: a population-based study. Lancet.

[2] Carabello B, Paulus W (2009). Aortic stenosis. Lancet.

[3] Baumgartner H, Falk V, Bax JJ (2017). 2017 ESC/EACTS guidelines for the management of valvular heart disease. The task force for the management of valvular heart disease. Eur Heart J.

[4] Baumgartner H, Hung J, Bermejo J (2017). Recommendations on the echocardiographic assessment of aortic valve stenosis: a focused update from the European Association of Cardiovascular Imaging and the American Society of Echocardiography. J Am Soc Echocardiogr.

[5] Eleid MF, Sorajja P, Michelena HI (2013). Flow-gradient patterns in severe aortic stenosis with preserved ejection fraction: clinical characteristics and predictors of survival. Circulation.

[6] Clavel M, Magne J, Pibarot P (2016). Low-gradient aortic stenosis. Eur Hear J.

[7] Clavel MA, Berthelot-Richer M, Le Ven F (2015). Impact of classic and paradoxical low flow on survival after aortic valve replacement for severe aortic stenosis. J Am Coll Cardiol.

[8] Lancellotti P, Magne J, Donal E (2012). Clinical outcome in asymptomatic severe aortic stenosis: insights from the new proposed aortic stenosis grading classification. J Am Coll Cardiol.

[9] Adda J, Mielot C, Giorgi R (2012). Low-Flow, low-gradient severe aortic stenosis despite normal ejection fraction is associated with severe left ventricular dysfunction as assessed by speckle-tracking echocardiography a multicenter study. Circ Cardiovasc Imaging.

[10] Cikes M, Solomon SD (2016). Beyond ejection fraction: an integrative approach for assessment of cardiac structure and function in heart failure. Eur Heart J.

[11] Lang RM, Badano LP, Mor-Avi V (2015). Recommendations for cardiac chamber quantification by echocardiography in adults: an update from the American Society of Echocardiography and the European Association of Cardiovascular Imaging. J Am Soc Echocardiogr.

[12] Haugaa KH, Smedsrud MK, Steen T (2010). Mechanical dispersion assessed by myocardial strain in patients after myocardial infarction for risk prediction of ventricular arrhythmia. JACC Cardiovasc Imaging.

[13] Prihadi EA, Vollema EM, Ng ACT (2019). Determinants and prognostic implications of left ventricular mechanical dispersion in aortic stenosis. Eur Heart J Cardiovasc Imaging.

[14] Kawakami H, Nerlekar N, Haugaa KH (2019). Prediction of ventricular arrhythmias with left ventricular mechanical dispersion: a systematic review and meta-analysis. JACC Cardiovasc Imaging..

[15] Risum N, Bhupendar A, Hansen TF (2015). Identification of typical left bundle branch block contraction by strain echocardiography is additive to electrocardiography in prediction of long-term outcome after cardiac resynchronization therapy. J Am Coll Cardiol.

[16] Nagueh SF, Smiseth OA, Appleton CP (2016). Recommendations for the evaluation of left ventricular diastolic function by echocardiography: an update from the American Society of Echocardiography and the European Association of Cardiovascular Imaging. J Am Soc Echocardiogr.

[17] Briand M, Dumesnil JG, Kadem L (2005). Reduced systemic arterial compliance impacts significantly on left ventricular afterload and function in aortic stenosis: implications for diagnosis and treatment. J Am Coll Cardiol.

[18] Zoghbi WA, Adams D, Bonow RO (2017). Recommendations for noninvasive evaluation of native valvular regurgitation: a report from the American Society of Echocardiography developed in collaboration with the Society for Cardiovascular Magnetic Resonance. J Am Soc Echocardiogr.

[19] Mehrotra P, Jansen K, Flynn AW (2013). Differential left ventricular remodelling and longitudinal function distinguishes low flow from normal-flow preserved ejection fraction low-gradient severe aortic stenosis. Eur Heart J.

[20] Clavel MA, Dumesnil JG, Capoulade R (2012). Outcome of patients with aortic stenosis, small valve area, and low-flow, low-gradient despite preserved left ventricular ejection fraction. J Am Coll Cardiol.

[21] Haland TF, Almaas VM, Hasselberg NE (2016). Strain echocardiography is related to fibrosis and ventricular arrhythmias in hypertrophic cardiomyopathy. Eur Heart J Cardiovasc Imaging.

[22] Haugaa KH, Grenne BL, Eek CH (2013). Strain echocardiography improves risk prediction of ventricular arrhythmias after myocardial infarction. JACC Cardiovasc Imaging.

[23] Rosa VEE, Ribeiro HB, Sampaio RO (2019). Myocardial fibrosis in classical low-flow, low-gradient aortic stenosis. Circ Cardiovasc Imaging.

[24] Klaeboe LG, Haland TF, Leren ISL (2017). Prognostic value of left ventricular deformation parameters in patients with severe aortic stenosis: a pilot study of the usefulness of strain echocardiography. J Am Soc Echocardiogr.

